# Virologic Failure of Protease Inhibitor-Based Second-Line Antiretroviral Therapy without Resistance in a Large HIV Treatment Program in South Africa

**DOI:** 10.1371/journal.pone.0032144

**Published:** 2012-03-13

**Authors:** Julie H. Levison, Catherine Orrell, Sébastien Gallien, Daniel R. Kuritzkes, Naishin Fu, Elena Losina, Kenneth A. Freedberg, Robin Wood

**Affiliations:** 1 Division of General Medicine Massachusetts General Hospital, Boston, Massachusetts, United States of America; 2 Division of Infectious Diseases, Massachusetts General Hospital, Boston, Massachusetts, United States of America; 3 Department of Medicine, Desmond Tutu HIV Center, Institute of Infectious Diseases and Molecular Medicine, University of Cape Town, Cape Town, South Africa; 4 Division of Infectious Diseases, Brigham and Women's Hospital, Boston, Massachusetts, United States of America; 5 Departments of Biostatistics and Epidemiology, Boston University School of Public Health, Boston, Massachusetts, United States of America; 6 Harvard University Center for AIDS Research, Harvard University, Boston, Massachusetts, United States of America; Boston University, United States of America

## Abstract

**Background:**

We investigated the prevalence of wild-type virus (no major drug resistance) and drug resistance mutations at second-line antiretroviral treatment (ART) failure in a large HIV treatment program in South Africa.

**Methodology/ Principal Findings:**

HIV-infected patients ≥15 years of age who had failed protease inhibitor (PI)-based second-line ART (2 consecutive HIV RNA tests >1000 copies/ml on lopinavir/ritonavir, didanosine, and zidovudine) were identified retrospectively. Patients with virologic failure were continued on second-line ART. Genotypic testing for drug resistance was performed on frozen plasma samples obtained closest to and after the date of laboratory confirmed second-line ART failure. Of 322 HIV-infected patients on second-line ART, 43 were adults with confirmed virologic failure, and 33 had available plasma for viral sequencing. HIV-1 RNA subtype C predominated (n = 32, 97%). Mean duration on ART (SD) prior to initiation of second-line ART was 23 (17) months, and time from second-line ART initiation to failure was 10 (9) months. Plasma samples were obtained 7(9) months from confirmed failure. At second-line failure, 22 patients (67%) had wild-type virus. There was no major resistance to PIs found. Eleven of 33 patients had a second plasma sample taken 8 (5.5) months after the first. Median HIV-1 RNA and the genotypic resistance profile were unchanged.

**Conclusions/ Significance:**

Most patients who failed second-line ART had wild-type virus. We did not observe evolution of resistance despite continuation of PI-based ART after failure. Interventions that successfully improve adherence could allow patients to continue to benefit from second-line ART therapy even after initial failure.

## Introduction

South Africa has the largest government-sponsored antiretroviral treatment (ART) program in the world [1]. Given the scarcity of salvage ART regimens in South Africa and other resource-limited settings and the high cost of second-line ART [2], rational use of second-line ART is critical.

International guidelines endorse boosted protease inhibitor (PI)-based combination ART as an efficacious strategy after failure of NNTRI-based first line ART [3]. Most adults are PI-naive at second-line ART initiation, PI resistance at failure of first-line NNRTI-based ART is uncommon [4], and PI-based second-line ART is highly potent [5]. Nevertheless, up to 40% of HIV-1 infected adults in South Africa develop confirmed virologic failure on second-line ART [6], [7], [8], [9].

In resource-limited settings such as South Africa, many unanswered questions remain about the contribution of drug resistance to second-line ART failure. They include uncertainties about the susceptibility of HIV-1 subtype C, which accounts for nearly half of global infections and the majority in South Africa [4], [10], [11]; effectiveness of ART delivery in public health clinics; and medication non-adherence.

Here, we investigated the extent to which drug resistance mutations contribute to second-line ART failure in a large community-based ART program in South Africa.

## Methods

### Ethics Statement

All study participants provided written informed consent. For participants age ≤18 years, an accompanying parent or guardian also provided written consent. Study procedures were approved by the University of Cape Town (Cape Town, South Africa) and the Partners HealthCare Human Research Committee (Boston, Massachusetts, USA).

### Study setting and population

The Gugulethu Clinic is an HIV referral center for a peri-urban township of Cape Town, South Africa. The clinic provides HIV-related care for more than 6,000 patients and serves an impoverished population of more than 300,000 individuals where the ante-natal HIV seroprevalence was 29% in 2006 [9], [12], [13]. Clinic demographic and clinical characteristics are consistent with other large ART roll-out programs in South Africa [10], [14]. Clinical data are prospectively maintained in an electronic database at the Desmond Tutu HIV Center. First-line ART consists of stavudine in the majority of cases, or zidovudine, with lamivudine and either efavirenz or nevirapine. Second-line ART includes zidovudine, didanosine, and lopinavir/ritonavir as per national protocol [15]. Consistent with national ART guidelines, patients were eligible to switch to second-line ART if they have persistent observed HIV viremia (HIV RNA>1000 copies/ml) at 2 consecutive occasions 3 months apart, despite an adherence intervention [15]. Patients with virologic failure are generally continued on second-line ART.

### Study Design

Data from HIV-infected patients ≥15 years of age who had failed second-line therapy (2 consecutive HIV-1 RNA tests >1000 copies/ml) by October 1, 2009, were analyzed retrospectively using a cross sectional design. A single local laboratory performed HIV-1 RNA assays by branch DNA hybridization techniques (HIV-1 RNA 3.0 assay®; Bayer Healthcare, Leverkusen, Germany) and CD4-count by flow cytometry (FACS Count™, Becton, Dickinson and Company, Franklin Lakes, NJ, USA). Genotypic testing for drug resistance was performed on frozen plasma samples obtained closest to, and on or after the date of laboratory confirmed second-line ART failure. We used International AIDS Society-USA criteria to define drug resistance mutations [16]. Some patients who failed second-line ART had genotypic drug resistance results available from first-line ART failure [4].

### Viral sequencing from plasma

Viral RNA was extracted (QIAamp viral RNA minikit, QIAGEN Inc) from 140 µl of each plasma sample and HIV-1 protease (PR codons 1–99, HXB2 nucleotides 2254–2549) and reverse transcriptase (RT codons 1–343, HXB2 nucleotides 2550–3577) were amplified by a 1-step reverse transcription–polymerase chain reaction (PCR), followed by a nested-PCR using gene-specific primers, as described [17].

Population (“bulk”) sequencing was performed on resulting amplicons with the Taq Dye Deoxy Terminator Kit (Applied Biosystems, Inc.) and resolved on an ABI 3730 automated DNA sequencer. Sequence processing was done using the Sequencher program (Genecodes). Sequences were aligned to the HIV-1 subtype B reference strain HXB2 (GenBank accession no. K03455).

Each sample was amplified in duplicate. A phylogenetic tree, using the PhyML program and including all the sequences generated as well as HXB2 and NL4-3 corresponding sequences, was used to confirm specimen identity and the absence of cross-contamination as an adjunct to the PCR negative control.

### Statistical Analysis

Mean values were reported with standard deviations (SD). Demographic and laboratory characteristics were examined for association with drug-resistant virus at second-line ART failure. We used Fisher's exact test to compare the association of categorical variables, age dichotomized at the mean (≤35 or >35 years), and HIV-1 RNA (≤10,000 copies/ml and >10,000 copies/ml) with the outcome. Statistical tests were two-sided with a significance level of 0.05. All statistical analyses were performed using SAS 9.1 software (Cary, North Carolina).

## Results

**Figure 1:**
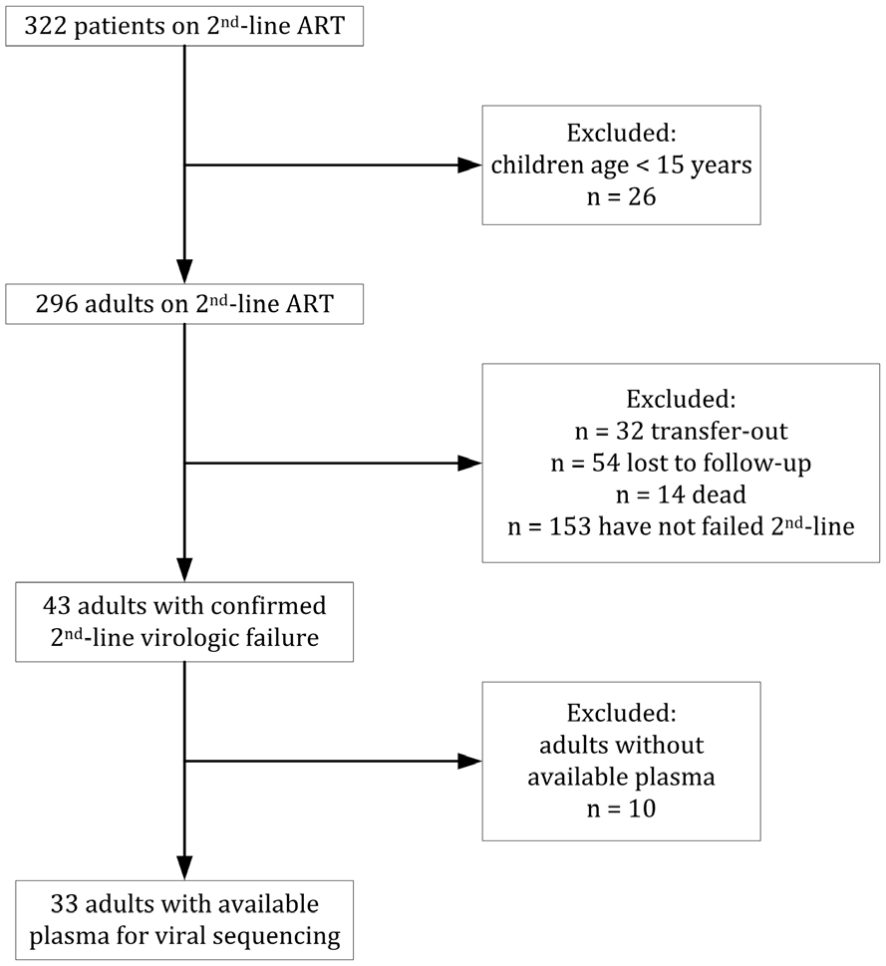
Study flow diagram.

Of 322 HIV-infected patients receiving second-line ART, 43 were adults (≥15 years) with confirmed virologic failure (Figure 1). Of these, 33 had plasma available for viral sequencing. No differences in age (p = 0.19), sex (p = 0.66), or viral load (p = 0.83) at second-line ART failure were found when comparing the 10 adults without plasma available for sequencing to the analyzed cohort. Of the 33 adults, mean (SD) age was 34 (8) years; 28 (85%) were female. HIV-1 RNA subtype C predominated (n = 32, 97%). Thirty-two of 33 individuals had available first-line treatment history. The majority (n = 30; 94%) received a stavudine-lamivudine based regimen; the remainder received zidovudine-lamivudine. Similarly, most patients received efavirenz as the NNRTI component (n = 24; 75%), and the rest received nevirapine. Mean (SD) duration on ART prior to initiation of second-line treatment was 23 (17) months. The median time between confirmatory HIV viral loads on second-line ART was 7 months. Mean time from second-line ART initiation to failure was 10 (9) months. Plasma samples available for analysis were obtained a mean of 7 (9) months from confirmed second-line failure.

At second-line ART initiation, median CD4 count was 210/µl [IQR 108–310/µl] and median HIV-1 RNA was 4.1 log_10_ copies/ml [IQR 3.5–4.7 log_10_ copies/ml], which increased to a median HIV-1 RNA of 4.6 log_10_ copies/ml [IQR 4.1–5.1 log_10_ copies/ml] at second-line failure.

At the first sample collection at second-line failure, virus from 22 patients (67%) was wild-type or lacked major drug resistance mutations (Table 1). Among samples with resistance mutations, NRTI resistance mutations were rare. Samples from 11 patients (33%) had major NNRTI resistance; of these, 9 patients had virus with only major NNRTI resistance and 2 patients had virus with both NRTI and NNRTI resistance. The most common NNRTI mutation was K103N (n = 5, 15%) found alone in 4 patients and with Y181C in another patient. Both Y181C (n = 3, 9%) and G190A (n = 3, 9%) were present with other resistance mutations. P225H (n = 1, 3%) occurred without other NNRTI resistance mutations. Resistance mutations that may reduce susceptibility to etravirine were assessed and were infrequent (Table 1).

**Table 1:** Distribution of genotypic drug resistance mutations for patients with virologic failure on second-line ART in a large ART roll-out program in South Africa.

Item	No. of patients (Total n = 33)	% of total	95% CI Low	95% CI High
I. No major mutations	22	67	51	83
II. NNRTI resistance mutations only†	9	27	12	43
One mutation				
K103N	4	12	1	23
P225H	1	3	0	9
G190A§	1	3	0	9
Y181C§	1	3	0	9
Two mutations				
K103N, Y181C	1	3	0	9
V106M, V179D§	1	3	0	9
III. NRTI and NNRTI resistance mutations	2	6	0	14
L74V, M184V (NRTI)G190A (NNRTI)	1	3	0	9
T69D, D67DG* (NRTI)Y181C, G190A (NNRTI)	1	3	0	9

ART: antiretroviral treatment. PI: protease inhibitor. NRTI: nucleoside reverse transcriptase inhibitor. NNRTI: non-nucleoside reverse transcriptase inhibitor.

* Thymidine analog mutation.

† Mutations at codons L100I, V108I, Y188C, were not detected.

§ Mutations reducing etravirine susceptibility. Others found in the cohort include V90I, K101E/P, V106I, and V179D. The A98G, L100I, Y181I/V, and M230L mutations were not detected.

Mutations classified as “minor” PI resistance mutations by the IAS-USA were detected in 30 of 33 (91%) individuals; no major PI resistance mutations were found. The most frequent minor PI resistance mutations included K20R (n = 9, 27%); M36I (n = 30, 91%), L63P (n = 14, 42%), H69K (n = 32, 97%), and I93L (n = 31, 94%). Less commonly found mutations included L10FV (n = 2, 6%) D60E (n = 5, 15%), and I62V (n = 3, 9%).

Ten individuals had HIV-1 genotypes from the time of first-line ART failure available. The same minor PI resistance mutations present at second-line ART failure were also present at first-line ART failure, prior to PI exposure. Virus from 8 individuals had M184V at first-line ART failure; in 7 individuals this mutation was undetectable at second-line failure. All samples in which the M184V mutation was detected at first-line failure also carried major NNRTI resistance mutations. Of the 9 individuals with virus containing major NNRTI resistance mutations at first-line failure, 6 retained virus with detectable major NNRTI resistance mutations at second-line failure. Samples from 2 individuals had thymidine analog mutations (TAMs) at first-line ART failure, which were not detected at second-line ART failure.

Eleven of 33 patients had a second sample for resistance testing taken 8 (5.5) months after the first sample at second-line failure. Median HIV-1 RNA was unchanged from the first sample at 4.5 log_10_ copies/ml [IQR 3.5–5.0 log_10_ copies/ml]. The genotypic resistance profile was also unchanged. Mean genotypic susceptibility score was 2.9 (±0.4) at second-line failure; this was unchanged for those with a repeat sample.

In patients aged 15–35 years, 29% had drug-resistant virus, compared with 42% of those aged >35 years, although this difference did not reach statistical significance. Thirty-one percent of individuals failing treatment with HIV RNA>10,000 copies/ml had drug-resistant virus, compared with 43% of individuals with HIV RNA≤10,000 copies/ml (p = 0.66).

## Discussion

In this large ART roll-out program in South Africa we identified patients with confirmed virologic failure on second-line ART. Despite the absence of major PI resistance at first-line ART failure, most individuals failed second-line ART quickly (e.g. within a mean of 10 months) and two-thirds failed with wild-type virus. Furthermore, while patients remained on second-line ART with continued virologic failure, drug resistance did not develop over the follow-up period.

Given the known potency of second-line ART regimens and the low frequency of drug resistant virus found, it appears that medication non-adherence is an important cause of second-line ART failure. While access to pharmacy refill, pill count, and patient-level pharmacokinetic data were not available, there are plausible patient-level, regimen-specific, and structural explanations for adherence problems on second-line ART. Drug toxicities related to lopinavir and didanosine and the buffered formulation of didanosine (the enteric-coated formulation was unavailable at the study site) likely hindered adherence [18], [19]. Social and structural obstacles to adherence can include inaccessible clinic location or lack of access to transportation, work/child-care responsibilities, and decreased health care provider to patient ratio as a consequence of the rapid growth in ART roll-out programs [20], [21], [22]. ART shortages, though not problematic at this site, can also be a challenge.

Most patients had substitutions in PR at sites that are known to be polymorphic in HIV-1 subtype C. These minor resistance mutations were also present at first-line ART failure in samples from the 10 patients who had samples available for sequencing from that time point. This observation confirms a prior genotype study from this clinic of HIV-infected individuals who were ART-naïve and who had failed first-line where the same polymorphisms in PR were present [4]. It is also consistent with other studies that performed genotypic analysis of individuals infected with HIV-1 subtype C who were ART-naïve [23], [24], [25], [26]. The impact of these polymorphisms has been debated, without clear clinical evidence that they affect drug susceptibility [27], [28].

Despite the absence of NNRTI exposure on second-line ART, virus from almost 30% of patients had at least one major NNRTI resistance mutation and virus from 15% had a mutation at the K103N codon, which suggests ongoing resistance to nevirapine and efavirenz. The persistence of NNRTI resistance is consistent with genotypic analysis of two other public sector South African cohorts of patients that failed PI-based ART [29], [30]. The low frequency of etravirine-associated mutations suggests excellent susceptibility to this next-generation NNRTI if it were to become available.

There are several therapeutic implications for the absence of NRTI resistance at second-line failure in this study. While other studies have suggested a higher rate of emergence of K65R at the time of stavudine-based ART failure in HIV-1 subtype C infection, we found that K65R did not appear after exposure to first-line stavudine or second-line didanosine; this may hold promise for the increasing use of tenofovir in resource-limited settings with predominance of nonsubtype-B HIV clades [31], [32]. M184V mutation and TAMs became undetectable supporting the hypothesis that lack of drug exposure due to ART non-adherence was the most likely cause of ART failure. In addition, consideration of archived resistance mutations is important in selection of second-line ART [33].

Females represented 85% of the patients whose viruses were sequenced, which is slightly higher than the distribution of females commencing second-line ART (75%)[9]. While the demographic and clinical characteristics of the cohort are largely consistent with other large ART programs, the small sample size may limit generalizability [34], [35]. Limited data were available on ART adherence, pharmacokinetic data, and contextual co-variates that may have an important impact on ART use and effectiveness. For example, those who receive treatment for other conditions, such as tuberculosis, may experience decreased levels of lopinavir/ritonavir if they are concomitantly taking rifampicin [36]. Because data on prior ART exposure, such as for prevention of mother-to-child transmission were not available, we limited the analysis to individuals who were documented as ART naïve at initiation of first-line ART in the clinical record. This study did not test for minority drug-resistance variants that are not detected by conventional genotypic testing but contribute to ART failure [37].

The low frequency of drug resistance to boosted-lopinavir at the time of virologic failure in this large ART-roll out program in Cape Town confirms other studies in Johannesburg and Soweto, South Africa, as well as in North America and Europe [29], [30], [38], [39]. *In vivo* resistance data suggest that the accumulation of accessory mutations in PR occurs rapidly only after major protease resistance mutations are established [40]. The absence of new PR mutations over a short interval of virologic failure on PI-based second-line ART is consistent with this observation.

In those who experience virologic failure on second-line ART in South Africa, ART failure occurs quickly and most often with wild-type virus. However, rapid development of resistance does not occur. Interventions that successfully improve adherence could allow patients to continue to benefit from second-line ART therapy even after initial failure.
